# Normal Bias in the Direction of Fetal Rotation Depends on Blastomere Composition during Early Cleavage in the Mouse

**DOI:** 10.1371/journal.pone.0009610

**Published:** 2010-03-10

**Authors:** Richard L. Gardner

**Affiliations:** Department of Biology, University of York, York, United Kingdom; Katholieke Universiteit Leuven, Belgium

## Abstract

Interest in establishing the basis of left/right asymmetry during embryogenesis has burgeoned in recent years. Relevant studies in mammals, focused largely on the mouse, have revealed involvement of a variety of genes that are common to the process in other animals. In the mouse, lateral differences in gene expression are first evident late in gastrulation when directional rotation of nodal cilia has been implicated in effecting the normally very strong bias in handedness. Reconstructing cleavage stages with correspondingly positioned blastomeres from appropriate numbers of conceptuses with similar division planes provides a way of testing whether they differ in potency without the confounding effects of reduced cell number. In a study using this strategy, 4-cell stage conceptuses reconstructed from blastomeres produced by equatorial as opposed to meridional second cleavage were found to be compromised in their ability to support normal development. Here, in more refined reconstructions undertaken at both the 4- and 8-cell stage, no significant impairment of development to the 9^th^ or 12^th^ day of gestation was found for products of equatorial second cleavage or their 8-cell stage progeny. Most surprisingly, however, a significant increase in reversal of the direction of axial rotation was found specifically among fetuses developing from conceptuses reconstructed from 8-cell stage progeny of products of equatorial second cleavage. Hence, manipulations during early cleavage some 6 days before fetal asymmetries are first evident can perturb the normally very strong bias in specification of a facet of left-right asymmetry.

## Introduction

**Figure 1:**
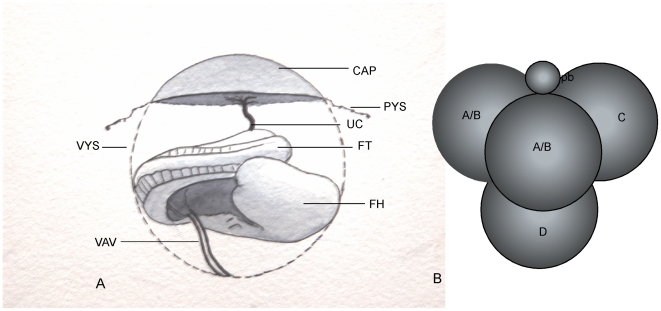
Asymmetries between fetus and rest of conceptus and shape of regular tetrahedral 4-cell stage. A). Diagram of a rotated fetus *in situ* with the parietal yolk sac (PYS) reflected back to the base of the chorio-allantoic placenta (CAP). The right side of the fetus faces towards the placenta, as does the umbilical cord (UC). Its tail (FT) is curled back against the right side of the trunk and head (FH), while the vitelline artery and vein (VAV) connect to the visceral yolk sac (VYS) from the left side of the still open abdomen. B) Diagram of a regular tetrahedral 4-cell stage conceptus showing the disposition of the products of meridional (A/B blastomeres) versus equatorial (C and D blastomeres) second cleavage relative to the second polar body (pb).

**Figure 2:**
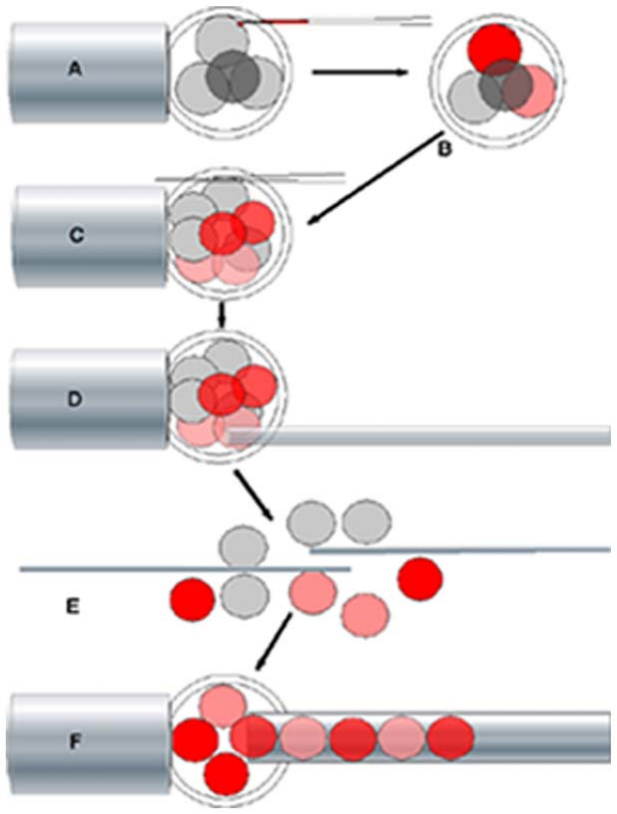
Reconstruction of 8-cell stage conceptus. Reconstruction of 8-cell stage conceptus from progeny of the products of E versus M second cleavage. A) One blastomere at the 4-cell stage, typically the product of E second cleavage that is remote from the 2^nd^ PB, was labelled with DiI. B) This was followed by short-term culture to encourage spread of the label to the sister blastomere and specimens showing spread selected for culture to the 8-cell stage. C) A long hairline slit was then made in the ZP before conceptuses were incubated for up to 35 min. in calcium-free medium plus EGTA before being returned to standard medium. D) Expulsion of fluid from a narrow-tipped pipette inserted through the slit in the ZP was then used to expel the blastomeres whose separation was completed with a pair of fine glass needles (E). F) Finally, the blastomeres isolated from several dissociated conceptuses were sorted into DiI-positive versus negative pools for injection into evacuated ZP. Strongly versus weakly positive blastomeres were separated for some E reconstructions.

The earliest lateral differences in gene expression in the mouse have been detected in Hensen's node, and seem to depend on the coordinated activity of nodal cilia [1]. They extend to the lateral plate mesoderm early in somitogenesis, and are followed shortly by rotation of the fetus about its antero-posterior axis. This axial rotation is almost invariably counter-clockwise in murine rodents [2], [3], typically resulting in the tail, umbilical cord and chorioallantoic placenta lying to the right of the fetus, and vessels of the yolk sac to its left (Fig. 1A). Although axial rotation begins shortly after lateral differences in gene expression are first seen, its direction bears a variable relationship to left-right asymmetries within the fetus [4]–[6]. Reconstructing early cleavage stage mouse conceptuses from appropriate numbers of ‘corresponding’ blastomeres taken from conceptuses with similar patterns of cell division avoids problems inherent in potency testing isolated blastomeres [7], [8]. Using this strategy, the products of equatorial (E) second cleavage (Fig. 1B) were found in one study to be compromised in development relative to those of meridional (M) second cleavage [9]. However, conceptuses were repeatedly removed from culture for examination by fluorescence microscopy and reconstructed in a denuded state rather than within the zona pellucida. Here, more refined reconstruction experiments have been undertaken (Fig. 2) which, while failing to show any significant difference in the pre- or post- implantation developmental potential of E versus M lineage blastomeres, revealed an effect of cellular composition at the 8-cell stage on the direction of fetal rotation.

## Results

Regardless of their cellular composition, conceptuses reconstructed at the 4- and 8- cell stage developed in vitro into morphologically normal late morulae or blastocysts in either all or nearly all cases (Table 1). Moreover, the rates of implantation and of normal postimplantation development of conceptuses reconstructed from the products of E second cleavage or their 8-cell stage progeny did not differ significantly from corresponding reconstructions from M blastomeres or sets of native blastomeres (Table 2).

**Table 1:** Rates of normal preimplantation development of reconstructed 4- and 8-cell stage conceptuses.

Stage/type of reconstruction	No. reconstructed	No. normal post-culture
4-cell native	20	19
4-cell M	43	43
4-cell E	44	43
8-cell native	50	49
8-cell M	148	146
8-cell E	157	157

**Table 2:** Rates of normal development of 4- and 8-cell stage conceptuses.

Stage & type of reconstruction	No. transferred to uterus *	No. implanted (% transferred)	No. of normal fetuses (%) implanted)
**4-cell native**	**17**	**17** (100)	**15** (88)
**4-cell M**	**43**	**37** (86)	**36** (97)
**4-cell E**	**39**	**33** (84)	**29** (88)
**8-cell native**	**33**	**30** (91)	**28** (93)
**8-cell M**	**107**	**93** (87)	**84** (90)
**8-cell E (total)**	**130**	**109** (84)	**99** (91)
**8-cell C**	**39**	**38** (97)	**34** (87)
**8-cell D**	**59**	**48** (81)	**43** (90)
**8-cell C&D**	**32**	**23** (72)	**22** (96)

**reconstructed from E or M versus native blastomeres.**

*Transferred to uterine horn yielding decidua.

**Figure 3:**
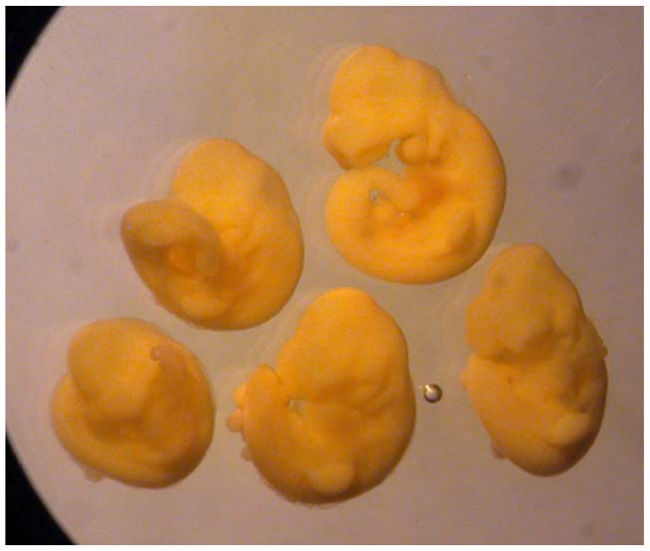
Litter of 5 fetuses of which two show clockwise rather than counter-clockwise axial rotation. A litter of five fetuses from 8-cell stage **E** reconstructions that have completed axial rotation and shown with their left side oriented uppermost. The tail is curled to the left of the trunk in the upper two and to the right in lower three.

An unexpected number of the more advanced fetuses developing from 8-cell stages reconstructed from the progeny of one or other or both products of the E second cleavage division had undergone clockwise (**c**) rather than counter-clockwise (**cc**) axial rotation so that both the tail and umbilical cord lay to the left rather than right side of the trunk (Fig. 3). Table 3 shows the incidence of **c** rotation among these fetuses compared with those from other experimental series and controls. The following points are evident from the table. The proportion of fetuses developing from 8-cell E reconstructions that had undergone c rotation (F) was significantly higher than among both in vivo controls (A) and those developing from corresponding 8-cell M reconstructions (G). The lack of a significant difference from control native reconstructions (***F*** vs ***B***) presumably reflects the limited size of the latter series since, were axial rotation affected by reconstruction *per se*, an increase should also have been evident in the 8-cell M experimental series. Unlike those of the M second cleavage division, the products of the E division could be identified readily through one member of the pair being remote from the second polar body (see Fig. 1B). Consequently, E and M reconstructions typically differed in being composed wholly of DiI-labelled versus unlabelled blastomeres, respectively. Hence, it is notable that the incidence of **c** axial rotation among fully DiI-labelled controls was also significantly lower than in the 8-cell E reconstructions (Table 3 ***F*** vs ***C***). Moreover, all 3 cases of **c** rotation encountered in the 4-cell stage M experiments (Table 3 ***E***) developed from conceptuses reconstructed entirely from unlabelled blastomeres. While cases of **c** rotation were encountered only among fetuses from M as opposed to E reconstructions at this earlier stage more data would be needed to establish whether this reflects a real difference.

**Table 3:** Experimental reconstructions at the 4- and 8- cell stage and controls.

CATEGORY OF FETUS	No. WITH TAIL TO LEFT OF TRUNK	TOTAL SCORED
**A**IN VIVO CONTROLS	2	134
**B**4- TO 8-CELL STAGES RECONSTRUCTED FROMNATIVE SETS OF BLASTOMERES	0	43*
**C**DIRECT DiI LABELLING OF ALL 4-CELL BLASTOMERES	0	56
**D**4-CELL STAGES RECONSTRUCTED FROMPRODUCTS OF **E** SECOND CLEAVAGE	0	30
**E**4-CELL STAGES RECONSTRUCTED FROM PRODUCTS OF **M** SECONDCLEAVAGE	3	33
**F**8-CELL STAGES RECONSTRUCTED FROMPROGENY OF PRODUCTS OF **E** SECOND CLEAVAGE	11**	99
**G**8-CELL STAGES RECONSTRUCTED FROM PROGENY OF PRODUCTS OF **M** SECOND CLEAVAGE	1	87

*31 of the 43 were reconstructed from conceptuses whose blastomeres were all directly labelled with DiI at the 4-cell stage.

**Of the 11, 4 (out of 34) were from reconstructions using only the progeny of the C blastomere, 5 (out of 43) from the progeny of D and 2 (out of 22) from mixed C and D progeny.

***F*** vs ***A*** P = 0.002|.

***F*** vs ***B*** P = 0.034| {P 0.05 = 0.013 using Bonferroni's correction}.

***F*** vs ***C*** P = 0.008|.

***F*** vs ***G*** P = 0.006|.

***E*** vs ***A*** P = 0.053|.

***E*** vs ***D*** P = 0,240|{P 0.05 = 0.025 using Bonferroni correction}.

In addition to the tail and umbilical cord, the vitelline blood vessels of the visceral yolk sac also showed reversal of normal asymmetry, emerging on the right rather than the left in all except one of 7 fetuses showing **c** rotation in which their sidedness was recorded. However, looping of the heart to the left rather than right was not seen any experimental or control fetuses examined in this study. Moreover, none of the 5 **c**-rotating fetuses that were scored on the 12 day of gestation when the stomach has shifted unambiguously to one side of the midline had this organ displaced to the right rather than, as normally, to the left.

## Discussion

The present results show that certain facets of L-R asymmetry are susceptible to perturbation during cleavage some 6 days before the processes leading to their establishment have been presumed to be initiated [10], [11]. They also confirm that specification of fetal visceral asymmetries can be dissociated from those defining relations between the fetus and the remainder of the conceptus, namely the direction of fetal rotation and consequent sidedness of its tail, umbilical cord and vitelline vessels. Moreover, they implicate early cell lineage in specification of the direction of axial rotation inasmuch that its normally very strong **cc** bias (<98% among in vivo controls) seems to require the presence of the progeny of products of the M second cleavage division. Thus, by the 8-cell stage blastomeres evidently differ in potential to specify a facet of patterning according to the distinctive planes of cleavage through which their lineages have originated. It was found in an earlier study that both pre-and post-implantation development was compromised in conceptuses that were wholly composed of E lineage blastomeres [9]. The possibility that this might account for the altered bias in direction of fetal rotation is not supported by present findings on comparative rates of development of the different classes of reconstructed conceptuses. The obvious disparity between findings in the two studies is difficult to explain. It is clear that blastomeres received harsher treatment in the former study. Moreover, conceptuses were reconstructed in a denuded state which has been shown to adversely affect pre-, and possibly also, post- implantation development [12]. However, such considerations do not account for why the E lineage specifically was affected which would seem to require that E and M lineage blastomeres differ in properties.

Not only do present findings challenge the claim that second cleavage planes are oriented randomly [13] but, more importantly, also the dogma that patterning is attributable entirely to positional cues, mechanical constraints or stochastic processes operating on sets of initially naïve blastomeres [14]–[17]. It does, however, accord with the view that pre-patterning is a facet of early development that mammals share in common with other metazoans [18]–[21]. It is noteworthy in this regard that the origins of L-R asymmetry have been traced back to cleavage in various organisms, including the frog, sea urchin, snail, and nematode worm [22]–[28].

## Materials and Methods

### Ethics Statement

All procedures on adult and fetal mice were undertaken as approved by the Oxford University Zoology Department Ethical Review Committee and with the authority of a Project Licence issued by the UK Home Office under the Animals (Scientific Procedures) Act 1986.

Conceptuses of the Pathology Oxford (PO) mouse strain were recovered at the 4-cell stage following natural mating of females selected for oestrus. Those with an approximately ‘regular tetrahedral’ form with the second polar body (2^nd^ PB) located at the intersection of 3 blastomeres (Fig. 1), were selected for manipulation, as shown in Fig. 2. Either the blastomere that was remote from the 2^nd^ PB (D in the notation of [7] or, in some cases, whichever of the three in contact with this body least overlapped D was labelled with the carbocyanine dye DiI (Molecular Probes, Eugene, Oregon). This least overlapping blastomere was shown previously to be one of the products of M second cleavage division in >90% of cases [7]. As controls for manipulation, further reconstructions were made using completely dissociated native sets of blastomeres from 4- or 8-cell stage conceptuses. Most of these had all blastomeres directly labelled with DiI, as did additional non-dissociated specimens. All conceptuses were then cultured for transfer to the uteri of pseudopregnant recipients at the late morula or blastocyst stage. Recipients were invariably killed between 8.5–11.5 days post coitum, both to ascertain rates of normal development and detect any morphological abnormalities. The stomach is displaced from the mid-line by 11.5 days, enabling an additional fetal visceral L-R asymmetry to looping of the heart to be scored whilst the tail still lies unambiguously to one side of the trunk.

Fisher's Exact test with the Bonferroni Correction for multiple comparisons were used to assess the significance of differences in incidence of reversed axial rotation.
